# Modeling the neuroimmune system in Alzheimer’s and Parkinson’s diseases

**DOI:** 10.1186/s12974-024-03024-8

**Published:** 2024-01-23

**Authors:** Wendy Balestri, Ruchi Sharma, Victor A. da Silva, Bianca C. Bobotis, Annabel J. Curle, Vandana Kothakota, Farnoosh Kalantarnia, Maria V. Hangad, Mina Hoorfar, Joanne L. Jones, Marie-Ève Tremblay, Jehan J. El-Jawhari, Stephanie M. Willerth, Yvonne Reinwald

**Affiliations:** 1 Department of Engineering, School of Science and Technology, Nottingham Trent University, Nottingham, UK; 2 Medical Technologies Innovation Facility, Nottingham Trent University, Nottingham, UK; 3 Department of Mechanical Engineering, University of Victoria, Victoria, Canada; 4 Division of Medical Sciences, University of Victoria, Victoria, BC Canada; 5 Centre for Advanced Materials and Related Technology (CAMTEC), University of Victoria, Victoria, BC Canada; 6 Department of Clinical Neurosciences, University of Cambridge, Cambridge, UK; 7 Department of Biosciences, School of Science and Technology, Nottingham Trent University, Nottingham, UK; 8 Department of Chemistry, University of Victoria, Victoria, BC Canada; 9 Neurosciences Axis, Centre de Recherche du CHU de Québec, Université Laval, Québec City, QC Canada; 10 Department of Molecular Medicine, Université Laval, Québec City, QC Canada; 11 Department of Biochemistry and Molecular Biology, The University of British Columbia, Vancouver, BC Canada; 12 Department of Neurology and Neurosurgery, McGill University, Montréal, QC Canada; 13 Institute On Aging and Lifelong Health, University of Victoria, Victoria, BC Canada; 14 Department of Clinical Pathology, Faculty of Medicine, Mansoura University, Mansoura, Egypt; 15 School of Biomedical Engineering, University of British Columbia, Vancouver, BC Canada

**Keywords:** Neuroimmune system, Alzheimer’s disease, Parkinson’s disease, Inflammation, Neurodegenerative diseases, Modeling

## Abstract

Parkinson’s disease (PD) and Alzheimer’s disease (AD) are neurodegenerative disorders caused by the interaction of genetic, environmental, and familial factors. These diseases have distinct pathologies and symptoms that are linked to specific cell populations in the brain. Notably, the immune system has been implicated in both diseases, with a particular focus on the dysfunction of microglia, the brain’s resident immune cells, contributing to neuronal loss and exacerbating symptoms. Researchers use models of the neuroimmune system to gain a deeper understanding of the physiological and biological aspects of these neurodegenerative diseases and how they progress. Several in vitro and in vivo models, including 2D cultures and animal models, have been utilized. Recently, advancements have been made in optimizing these existing models and developing 3D models and organ-on-a-chip systems, holding tremendous promise in accurately mimicking the intricate intracellular environment. As a result, these models represent a crucial breakthrough in the transformation of current treatments for PD and AD by offering potential for conducting long-term disease-based modeling for therapeutic testing, reducing reliance on animal models, and significantly improving cell viability compared to conventional 2D models. The application of 3D and organ-on-a-chip models in neurodegenerative disease research marks a prosperous step forward, providing a more realistic representation of the complex interactions within the neuroimmune system. Ultimately, these refined models of the neuroimmune system aim to aid in the quest to combat and mitigate the impact of debilitating neuroimmune diseases on patients and their families.

## Background

Neuroinflammation or central nervous system (CNS) inflammation is present in neurodegenerative disorders. It can contribute to the progression of pathologies like Alzheimer’s disease (AD), Parkinson’s disease (PD), amyotrophic lateral sclerosis, Huntington’s disease, and multiple sclerosis [1]. In these pathologies, not only is the CNS affected, but alterations of the immune system have also been shown. Under healthy conditions, the immune system is involved in building, maintaining, and repairing the CNS, particularly through extracellular debris’ phagocytosis and production of neurotrophic factors. In neurodegenerative diseases, the immune system sustains neuroinflammation, leading to a pathology progression [2]. This review focuses on AD and PD, complex neurodegenerative disorders that have a profound impact on individuals and society. The interaction between neuroinflammation, immune dysregulation, and the accumulation of abnormal protein aggregates, such as amyloid-beta and Tau in AD and α-synuclein in PD, is complex and has remained largely elusive. Modulating the immune system might offer a possible path toward finding effective treatments for these diseases [3, 4]. Thus, a comprehensive understanding of the pathophysiology of AD and PD, involving complex interactions between the immune system and the CNS, is paramount for treatments, and for the generation of functional models to study these conditions. This review aims to highlight the significance of understanding the pathophysiology and biology of AD and PD and their connections to the immune system. By unraveling the underlying mechanisms and pathways involved in these diseases, we can gain novel insights into potential therapeutic targets and develop strategies for early detection and intervention.

## Introduction

### Alzheimer’s disease and Parkinson’s disease etiology

**Fig. 1:**
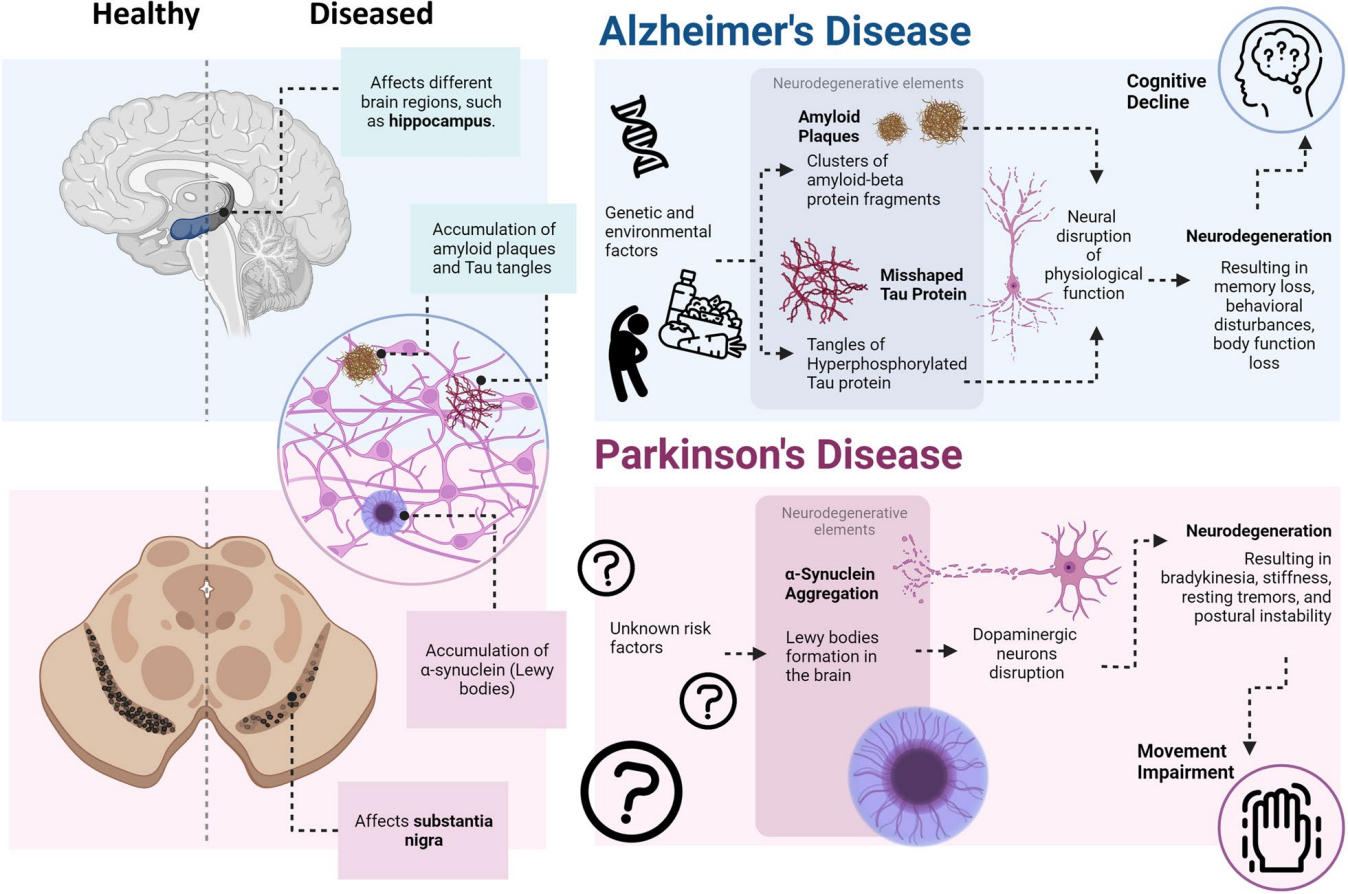
Pathophysiology of Alzheimer’s disease and Parkinson’s disease. The schematic shows the risk factors and features associated with both disease conditions. Image created with BioRender.com

**Fig. 2:**
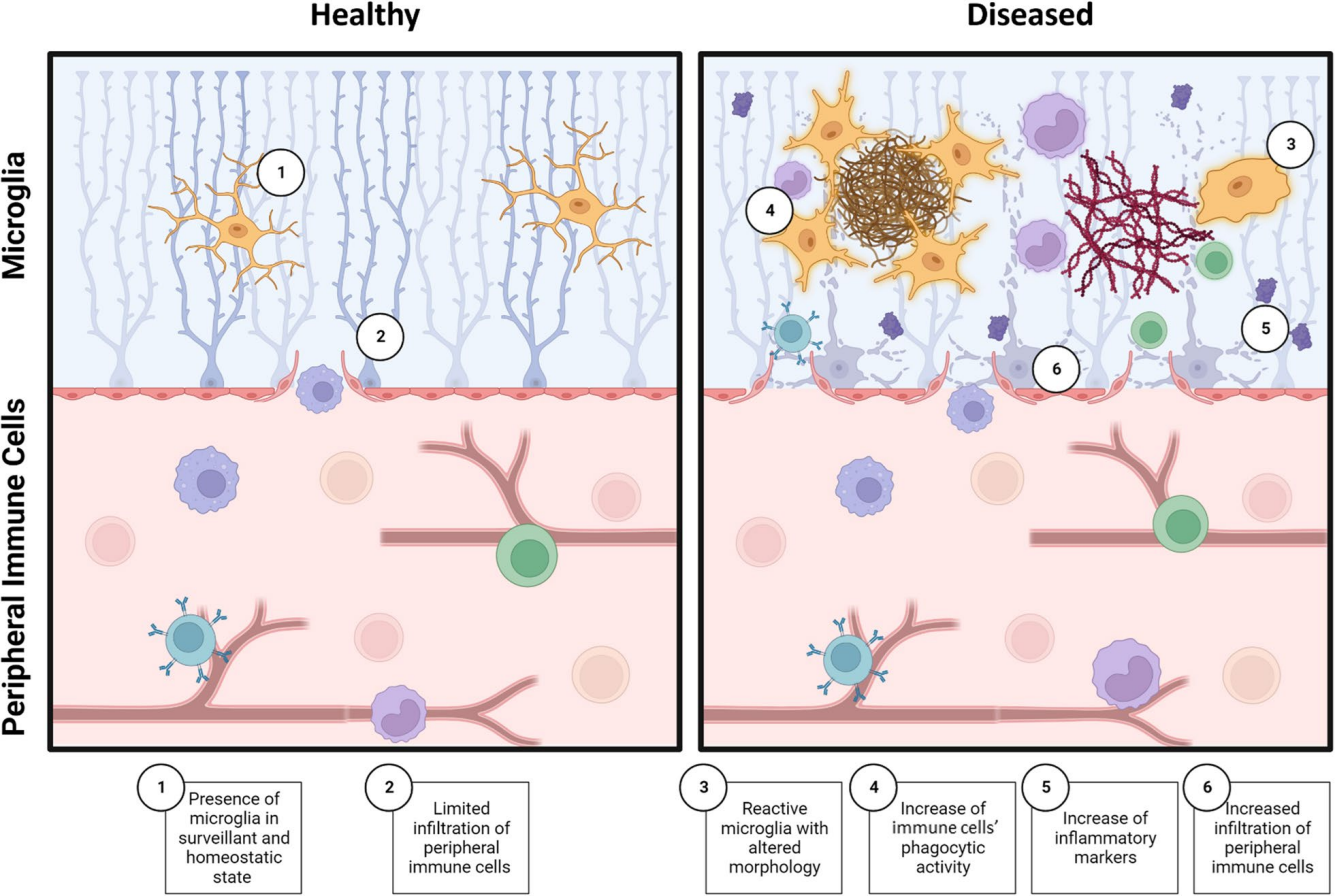
Comparison of healthy versus pathological neuroimmune system: in healthy neuroimmune system (1) microglia are in a homeostatic and surveillant state, (2) with limited infiltration of peripheral immune cells into the central nervous system. In the pathological neuroimmune system, (3) microglia become reactive and display an altered morphology, with increase of (4) phagocytosis, (5) inflammatory markers and (6) peripheral immune cell infiltration. Image created with BioRender.com

AD and PD are among the most prevalent neurodegenerative conditions, inflicting a detrimental outcome on individuals and society worldwide. The impact of these diseases is aggravated by the lack of effective treatments, resulting in increased morbidity and mortality, and representing consequently a major public health challenge [5]. The global socioeconomic bearing of neurodegenerative diseases is increasing significantly as life expectancy rises. Despite extensive research, the limitations of current approaches can be attributed to the complexity of the cellular and molecular mechanisms underlying these diseases. Common pathophysiological mechanisms include abnormal protein dynamics, oxidative stress, mitochondrial dysfunction, DNA damage, neurotrophin dysfunction and exacerbated or altered neuroinflammatory processes (Fig. 1) [6–8]. Recently, the discovery of increased circulating levels of pro-inflammatory markers in AD and PD patients suggested a strong interplay between various neuroimmune and inflammatory mechanisms [9, 10]. Therefore, it is essential to understand these pathologies and explore their mechanistic underpinnings, particularly via the development of complete and detailed in vitro models (Fig. 2).

#### Alzheimer’s disease

The most common type of dementia in elderly is AD, which is a non-reversible degeneration of the brain that impairs memory, cognition, personality, and other functions. The most common cause of death in patients with severe AD is aspiration pneumonia, while for the milder cases, it is myocardial infarction [11]. AD risk factors include genetic and environmental factors, such as chronic stress, diet, exercise, smoking, traumatic brain injury, diabetes, and other medical conditions [6, 8, 12]. Although the causes of this disease are not fully understood, AD has been associated with neurodegenerative elements such as amyloid plaques, fragments of amyloid-beta (Aβ) that cluster and disrupt neuronal functions. Aβ is not naturally found in the brain; its presence results from the pathological cleavage of amyloid precursor protein (APP) [13]. Despite the long-held belief in the amyloid cascade hypothesis, the association between amyloid deposits and AD remains complex and not as straightforward as initially thought. Studies suggest that short amyloid fragments can increase the membrane permeability to calcium ions and consequently disrupt the delicate balance of calcium homeostasis within the neuron [14]. Aβ accumulation was also associated with glucose metabolism and cognitive decline. In fact, Aβ tends to accumulate in brain regions with higher glucose metabolism and extensive plaque formation, leading to a gradual decline in glucose metabolism and cognitive function [15]. Aβ plaques’ accumulation in AD is associated with the apolipoprotein E (APOE) gene, particularly the APOE4 allele, which is a major risk factor for late-onset AD. Genes involved in amyloid precursor protein processing, such as APP, presenilin 1 and 2, are linked to early-onset familial AD [16]. Variants in genes related to inflammation and immune responses, like clusterin, complement receptor 1, and triggering receptor expressed on myeloid cells 2, also play a role in Aβ plaque formation [17, 18]. Additionally, neurofibrillary tangles, aggregates of hyperphosphorylated Tau protein, have been associated with AD. It was believed that Tau’s main function in physiological conditions was to stabilize microtubules in the axons of neurons. However, emerging evidence suggests that Tau’s physiological functions involve the regulation of phosphorylation-based signaling pathways [19]. When Tau undergoes hyperphosphorylation and aggregation, these functions are disrupted, resulting in a compromised axonal transport. This, in turn, exacerbates the neurodegenerative environment [19, 20]. Although the exact causes of the misshaping of Tau proteins are not fully understood, they have been linked to various factors, including a genetic variant in APOE gene [21]. Currently, there are no dependable techniques available to predict the onset of AD, resulting in individuals’ unawareness of their risk of developing the condition until symptoms manifest [22]. Discerning the manifestations of normal aging, dementia, and AD can be challenging because symptoms can overlap. During aging, the immune system weakens which is often accompanied by the prevalent occurrence of chronic low-level inflammation, referred to as inflammaging. Mild cognitive impairment (MCI) serves as a crucial stage in this spectrum, where individuals experience cognitive changes beyond normal aging but do not meet the criteria for dementia or AD. Identifying and distinguishing MCI from other cognitive conditions becomes essential for timely intervention and management to potentially slow down cognitive decline [23]. Multiple studies have demonstrated that higher levels of peripheral inflammatory markers in mid- to late-life are associated with an increased risk of dementia and cognitive decline. These events can have complex effects on the neuroinflammatory response in AD, potentially leading to immunosuppressive or anti-inflammatory environments within the brain. As described in depth in Sect. 2.2.1, peripheral inflammation and dysregulation of the immune system in the CNS contribute to the pathogenesis of AD [24, 25].

#### Parkinson’s disease

PD is a neurological disease, mostly recognized as a movement-related disorder, with deep grey matter degeneration caused by neuronal loss in the substantia nigra, resulting in a lack of dopamine in the basal ganglia [6–8, 26]. Along with non-motor symptoms such as insomnia, constipation, dementia, and cognitive and olfactory impairments, the well-described motor symptoms include bradykinesia, stiffness, resting tremors, and postural instability [6–8, 12, 27]. PD’s development and association with the death of dopaminergic neurons in the substantia nigra remain unclear [28]. Evidence suggests a link to Lewy bodies, abnormal protein deposits primarily composed of aggregated α-synuclein (α-syn). α-syn, a protein normally present in presynaptic terminals, regulates neurotransmitter release [29, 30]. In neurodegenerative diseases, the imbalance between the production and clearance of α-syn can lead to its misfolding and accumulation into Lewy bodies [31, 32]. Several genetic variants have been associated with α-syn aggregation and increased risk of PD. For instance, mutations in the α-syn gene (*SCNA*) can lead to abnormal protein folding and aggregation. Additionally, variations in genes involved in α-syn metabolism and clearance, such as leucine-rich repeat kinase 2 gene (*LRRK2*), glucocerebrosidase gene (*GBA*), and parkin RBR E3 ubiquitin-protein ligase gene (*PRKN*), have also been implicated in PD pathogenesis [33]. A recent study examined the impact of Lewy bodies on neurons in the nucleus basalis of Meynert. In patients displaying PD symptoms, the presence of Lewy bodies was associated with mitochondrial damage and neuronal loss. Moreover, Lewy bodies may also be involved in sequestering damaged mitochondria and/or α-syn oligomers, providing protection against their harmful effects [34]. The α-syn aggregates in the brain interfere with the normal functioning of dopaminergic neurons, thus leading to motor impairments, accompanied by depression, anxiety, and apathy [35, 36]. This is attributed to the spread of α-syn pathology to regions beyond the substantia nigra, including cortical areas involved in cognition, mood regulation, and emotional processing [36]. Efforts to predict PD have led to the exploration of potential biomarkers associated with key molecules involved in the disease. Lin et al. investigated the levels of α-syn, Aβ-42, Tau, and nuclear DNA in peripheral plasma, along with regional gray matter volumes, in patients with idiopathic PD. The findings revealed three distinct patterns of gray matter volume atrophy corresponding to different stages of PD. Additionally, the study unveiled correlations between proteinopathy, inflammation markers, gray matter volume patterns, and cognitive performance in PD [37]. While PD can occur at any age, the prevalence of the disease rises significantly in elderly people. The natural decline in the function of the dopaminergic system, accompanied by age-related processes, such as accumulation of oxidative stress, impaired mitochondrial function, altered protein handling mechanisms, as well as altered immune function and neuroinflammation have been implicated in the pathogenesis of PD [38].

### Immune system associated with Alzheimer’s and Parkinson’s diseases

In the CNS, components from the innate and adaptive immune system can be found. Microglia are the resident innate immune cells, which originate from the embryonic yolk-sac and colonize the brain in early development. These cells play diverse roles in maintaining the function, plasticity, and integrity of the CNS. Additionally, microglia are actively involved in maintaining homeostasis of the CNS upon various challenges. They contribute significantly to neurogenesis, synapse formation, modification and pruning, vasculature maintenance and repair, axonal myelination, and other essential activities that support CNS activity and health [39, 40]. However, these cells can become reactive, releasing excessive amounts of pro-inflammatory and toxic molecules and perform aberrant phagocytosis that cause synaptic loss and neurodegeneration when exposed to chronic challenges. Over the course of aging, microglia can also become dystrophic and senescent, displaying altered morphology, transcriptome, proteome, lipidome and consequently, impaired functions [41]. With age-associated changes, microglia become less efficient at surveying the brain parenchyma, remodeling synapses, and clearing away damaged cells and toxic debris [42]. Other myeloid cells include monocytes and macrophages, specialized innate immune cells responsible for phagocytosis, release of cytokines and T cells antigen-presentation [43]. In addition, adaptive immunity is the ability to withstand infection and tissue damage caused by specific antigens after previous exposure. This protection comes from adaptive responses from lymphocytes, a bone-marrow lineage derived from hematopoietic stem cells, which are found in the bloodstream. Multiple subtypes of lymphocytes collaborate to coordinate adaptive immune response. For instance, the effector T cells (Teffs), particularly T helper (Th, CD4 +) cells, recognize foreign antigens and coordinate their response with other Teffs, including cytotoxic T cells (CD8 +), regulatory T cells (Tregs) and B cells [25]. CD8^+^ cells are primarily responsible for recognizing and eliminating target cells, through the release of cytotoxic molecules, such as perforin and granzymes [44]. By contrast, T_regs_ are specialized subsets of CD4^+^ T cells that have immunosuppressive functions to avoid self-antigens response [45]. Lastly, B cells, also bone-marrow derived, are a lymphocyte type specialized in antibody production and antigen presentation, to neutralize foreign pathogens and resist infections [46].

‘Neuroinflammation’ is a complex phenomenon referring to the inflammatory response of the CNS to various insults, such as injury, infection, or neurodegeneration [47, 48]. While inflammation is a normal protective response of the immune system, chronic or excessive inflammation can have detrimental effects on the CNS [47]. CNS exacerbated inflammation is implicated in various neurological disorders, including AD and PD.

#### Neuroinflammation and immune dysfunction in Alzheimer’s disease

In AD, CNS over-inflammation is marked by numerous factors that work together to drive the disease progression. Dysregulated levels of various anti- *versus* pro-inflammatory cytokines, the phenotypic transformation of immune cells such as microglia in the CNS, and the recruitment of macrophages and lymphocytes from the periphery, contribute to synaptic loss, which is the most accepted pathological correlative of the subsequent cognitive impairment [47].

Among the pathological mechanisms driving AD, the elevated pro-inflammatory cytokine production during healthy middle-age, taking place outside the CNS by endothelial cells and adipocytes upon injury, or inside the CNS via neurons, astrocytes and other immune cells upon inflammation or infection, can be associated with an increased risk of developing dementia or AD later in life. Elevated levels of interleukin (IL)-6 and -1β, as well as tumor necrosis factor alpha (TNF-α) were found in elderly individuals’ blood samples who had a first-degree relative with dementia. High blood cytokine levels were also associated with a greater risk of developing mild cognitive impairment (MCI). This indicates that pro-inflammatory cytokines may contribute at a preclinical stage to the brain chronic inflammation found in AD cases [49, 50]. Studies on the effect of cytokines on AD progression can be found in Table 1.

**Table 1:** Effects of innate and adaptive immune cells on Alzheimer’s disease progression

Experimental approach	Effects on cells	Effects on Alzheimer’s disease progression	Model or samples	References
Cytokines				
Effect of IL-1β on mouse primary neurons	Prostaglandin concentration increase, presynaptic glutamate release, postsynaptic N-methyl-d-aspartate receptor activation	Neurotoxicity, oxidative stress, and inflammation, leading to neuronal dysfunction and synapse loss	Mouse cells with prostaglandin-endoperoxide synthase 2 knock out	Zhou et al*.* [61]
Effect of IL-1β on mouse primary neurons exposed to Aβ oligomers	Mitochondrial dynamics disrupted, mitochondrial fission, fusion, and transport comprised	Increase of synapse loss and memory impairment	Mouse cells from hippocampus	Batista et al*.* [62]
Microglia				
IBA1^+^ and CX3CR1^GFP^ cells (immunohistochemistry and confocal microscopy)	Less ramified microglia when near Aβ plaques and compared to microglia from wild-type mice	Strong morphological changes in plaque-associated microglia Plaque-distant microglia showed minor changes	3.5 to 5.5-month-old TgCRND8^wt/wt^; CXC3CR1^GFP/wt^ and TgCRND8^wt/tg^; CXC3CR1^GFP/wt^	Plescher et al. [63]
IBA1^+^ cells (immunohistochemistry and confocal microscopy)	Dystrophic microglial morphologies, shortened cell arborization and cell soma size’s enlargement in the cerebral cortex	AD did not affect microglial density, but impaired morphology, with decreased ramified microglia in AD cases	*Post-mortem* samples of individuals with AD (44) and healthy controls (32)	Franco-Bocanegra et al. [64]
IBA1^+^ and P2Y12^+^ cells (fluorescence-activated cell sorting and immunostaining)	SPP1 upregulation induced microglial phagocytic states in the hippocampus and enhanced synaptic engulfment by microglia	SPP1 is required for microglia to engulf synapses. Absence of Spp1 expression prevents synaptic loss suggesting crosstalk between perivascular cells and microglia	3- to 4-month-old APP/ SPP1^tm1(tdTomato)Msasn^ and APP^WT^ *versus* APP^NL−F^ mice wild-type mice	De Schepper et al. [54]
Peripheral macrophages and monocytes				
Plasma levels of monocyte chemoattractant protein-1 (MCP-1)	Highest MCP-1 concentration in AD patients compared to MCI and controls	MCP-1 dysregulated peripheral chemoattractant factors in AD contribute to inflammation	Individuals with AD (310), MCI (66) and healthy subjects (120). Age 70–80 years	Lee et al. [65]
Plasma levels of isolated monocytes/macrophages (CD14 + and CD16 −/+)	Different subsets of monocytes were found classical (CD14 + + CD16 −), intermediate (CD14 + + CD16 +) and non-classical (CD14 + CD16 + +). Intermediate subset was higher in MCI and AD groups	Hyperactivity of monocytes and macrophages during MCI contributes to AD progression. Circulating monocytes from AD patients produced significantly higher levels of free radicals compared to healthy subjects	Comparison among individuals with AD (14), subjective memory complaints (10), MCI (14), and healthy subjects (15) Age range of 60–85 years	Munawara et al. [58]
T cells				
T helper 1 and Th17 Aβ-reactive IBA1^+^ cells (immunostaining); CD4^+^CD25^+^ and CD4^+^CD25^−^ flow cytometry; hippocampal transcriptional analysis	Th1 and Th17 Aβ-reactive facilitated Aβ deposition and suppressed T cells activity APP/PS1/Aβ-Th1 mice showed an increased IBA1^+^ microglial cells density compared to controls. IBA1^+^ displayed a processes shrinkage and a more amoeboid morphology	Administration of Aβ reactive Th1 and Th17 cells to APP/PS1 mice resulted in an acceleration of memory impairment and systemic inflammation, an increase in amyloid burden, heightened microglia reactivity, and intensified neuroinflammation	6-month-old APP/PS1 mice transfused with cloned lines of Aβ-Th1 and Aβ-Th17 cells compared to untreated and age-matched wild-type mice	Machhi et al. [60]
β-site APP-cleaving enzyme 1 (BACE1) (magnetic activated cell sorting of CD4 + T cells; flow cytometry analysis and western blotting for BACE1 expression)	BACE1 expression was higher in AD patients than healthy controls CD4^+^ T cells in HUBC mice exhibited higher levels of BACE1 expression. CD4^+^ T cells with elevated BACE1 expression showed increased production of interleukin-2	Overexpression of BACE1 potentiates CD4^+^ T cell reactivity in patients with AD	29 AD patients, 27 healthy controls. Age 53–82 years. 4-month-old HUBC (human ubiquitin C promoter) mouse model compared age-matched wild-type and 5 × FAD mice	Dai et al. [66]
T-effector cells (immunohistochemistry; transcriptome analysis of the hippocampus)	CD8^+^ cells infiltrated the hippocampus, cortex and corpus callosum of APP/PS1 and even aged wild-type	CD8^+^ invade the AD brain and control the expression of genes related to neurons and synapses in APP-PS1 mice	3- to 26-month-old APP/PS1 compared to age-matched wild-type mice	Unger et al. [67]

The microglial impact on AD pathology is complex and multifaceted [51]. During disease progression, physiological roles of microglia in surveillance, response to damage, and phagocytosis become impaired [52]. Microglia also contribute negatively to the spread of Aβ and Tau pathology. The mechanisms driving these different roles of microglia in AD pathology remain largely unclear, with microglial metabolic alterations being the main accepted hypothesis to explain these events [53]. A decreased microglial responsiveness to cues, and a reduced performance in their essential functions, such as surveillance, trophic support, and phagocytosis, are associated with the microglial changes present in the AD pathology [54]. Interestingly, a microglial state named “dark microglia” was recently found in mice and humans and is predominantly observed during aging and AD pathology. These cells exhibit a condensed and electron-dense cytoplasm and nucleoplasm, along with markers of oxidative stress, which classifies them as “dark” cells [55]. These cells portrait a hyper-ramified morphology, are immunoreactive for CD11b and triggering receptor expressed on myeloid cells 2 (TREM2), and weakly immunoreactive for conventional microglial markers, like P2Y12 and IBA1. These cells further associate with Aβ plaques and surround axon terminals and dendritic spines, and they have been found in many brain regions, including the hippocampus [55, 56]. Details of the latest studies on microglia can be found in Table 1.

As “inflammaging” confers an important risk of developing AD, the progressive release of pro-inflammatory mediators by monocytes and macrophages during aging is also associated with AD onset and progression [57]. When comparing AD patients with healthy patients, circulating monocytes from AD patients were found to produce significantly higher levels of free radicals compared to those of healthy subjects [58]. Inflammatory or chemoattractant cascades are also altered in AD. For instance, the monocyte chemoattractant protein-1 (MCP-1), during chronic inflammation, contributes to an overactivity and excessive recruitment of inflammatory cells, which leads to neuronal damage and cognitive decline [59]. Inflammation and aging are determining factors that affect innate and adaptive immune responses. Several studies demonstrated altered distributions and phenotypic transformation of T helper and cytotoxic T cells in the periphery, resulting in complex implications for AD progression [60]. Details of the latest studies on microglia cells, peripheral monocytes, macrophages, and T cells can be found in Table 1.

#### Neuroinflammation and immune dysfunction in Parkinson’s disease

Both peripheral and central inflammation have been widely associated with PD pathogenesis and progression. Key natural history studies have revealed associations of inflammation and immune cell activation with disease progression, particularly with risk of differential prognoses, such as higher risk of cognitive impairment and dementia and lower risk of motor dysfunction [68–70]. Genetic linkage studies and genome-wide association studies have linked PD risk with mutations in immune-related genes, such as HLA-DR [71–73]. Furthermore, PD shows pathological similarities to autoimmune diseases such as inflammatory bowel disease and genetic variants have been found to be shared between PD and other autoimmune conditions [74, 75]. In healthy individuals and early in the PD disease process, microglia are considered beneficial by clearing protein aggregates, releasing neurotropic factors, and performing synaptic pruning essential for learning and memory. However, microglia in the substantia nigra of PD patients were found to be dystrophic, releasing higher levels of pro-inflammatory cytokines IL-1β, IL-6, IL-12, TNF-α, s and inflammatory molecules such as ROS and nitric oxide [76–78]. These pro-inflammatory factors can have direct neurotoxic effects on the dopaminergic neurons (DAN); chronic exposure of neurons to these factors can lead to degeneration [79, 80]. Among other mechanisms of glial modulation, both microglia and astrocytes can be directly activated by α-syn, which is recognized by Toll-like receptor (TLR)-2 and TLR-4 on astrocytes and microglia. Upon recognition of α-syn, glia upregulates pro-inflammatory downstream pathways and secrete pro-inflammatory cytokines. In astrocytes, increased inflammasome activity is particularly notable [81]. In the periphery, both innate and adaptive immune cells are affected in PD; innate monocytes, macrophages and neutrophils secrete higher levels of pro-inflammatory cytokines. Like microglia, circulating monocytes and macrophages express higher levels of TLR-2 and TREM2. TREM2 stimulates microglial phagocytosis, maintains metabolic fitness, and suppresses the pro-inflammatory cytokine production. Adaptive immune dysfunction is likely to contribute to earlier symptoms of PD that occur before motor onset [82, 83]. T cells reactive to α-syn can be detected 10 years before motor diagnosis as well as later in the disease process [84]. In addition to their reactivity to α-syn, T cell numbers in PD patients were found to be widely dysregulated; CD3 + cells as a proportion of total leukocytes are lower, but there is a skew towards pro-inflammatory Th1 and Th7 subsets, with lower Th2 and T-reg cells. Concurrently, T cells are more primed for activation and release higher levels of pro-inflammatory cytokines, reflected by increased TNF-α, IL-1β, IL-2 and IL-10 in the serum of PD cases [85–89]. B cells are also implicated in PD pathogenesis, though their involvement is less well elucidated than the T cells. Higher autoreactive α-syn antibodies are detected in PD blood [90] and like T cells, the total B cell number is lower and there is a skew towards pro-inflammatory B cells. There is evidence of increased TNF-α and IL-6 production, higher ratio of IgG to other Ig molecules, indicative of antibody class switching and activated humoral response. Furthermore, B cells in PD blood express higher levels of antigen presentation molecules, suggesting increased support of T cell activation [91, 92]. In healthy individuals, the central and peripheral immune systems are structurally distinct, thanks to the presence of the highly specialized blood–brain barrier (BBB). In PD, the BBB is known to be leaky, allowing for a less-regulated passage of the peripheral immune cells into the brain parenchyma. When peripheral immune cells infiltrate, they can induce neurotoxicity and phenotypically transform microglia and astrocytes, worsening neuroinflammation [93].

## Current treatments and clinical trials for Alzheimer’s disease and Parkinson’s disease

One of the common drug targets in AD is acetylcholinesterase, an enzyme that is more active in patients with AD, leading to a lack of acetylcholine in the brain and hence, increased Aβ plaque production (Table 2). Lately, different research groups have focused on the development of drugs able to reduce the Aβ load in AD, primarily using monoclonal antibodies [94]. Among these, lecanemab was approved in January 2023, and aducanumab was granted conditional accelerated approval by the FDA in 2021. The drug will undergo phase 4 clinical trial, which will end in 2030 [95]. Details about these drugs can be found in Table 2.

**Table 2:** Drugs treating Alzheimer’s and Parkinson’s disease that have passed phase 3 clinical trials

Type of drug	Drug name	Company	Working principle	Side effects	References
Acetylcholinesterase inhibitors (AChE)	Aricept^®^ (donepezil)	Eisai Co. Ltd and Pfizer	AChE inhibitors avoid acetylcholine breakdown, restoring and improving the neurotransmitter activity	Diarrhea, nausea, and vomiting	[107]
	Exelon^®^ Patch (rivastigmine)	Novartis Pharmaceuticals Corporation			
	Razadyne™ (galantamine)	Johnson and Johnson			
Human immunoglobulin gamma 1 (IgG1) monoclonal antibody targeting Aβ	LEQEMBI™ (lecanemab)	Biogen	Binds to soluble Aβ protofibrils to prevent Aβ plaques	Amyloid Related Imaging Abnormalities (ARIA), flu-like symptoms, nausea, vomiting and variations in blood pressure	[108–110]
	Aduhelm™ (aducanumab)	Biogen	Binds to Aβ plaques in the brain	ARIA-edema, ARIA-hemosiderin deposition (ARIA-H) microhemorrhage and headache	[95, 111]
Glycogen synthase kinase 3 (GSK3β) inhibitor to restore gut microbiome	ANAVEX 2–73 (blarcamesine)	Anavex Life Science Corp	Sigma-1 receptor activator, involved in enhancing neuroplasticity and neural cells homeostasis	Dizziness, headache	[112, 113]
Dopamine replacement	Levodopa	AbbVie Inc	Catecholamine precursor converted to dopamine in the brain by DOPA decarboxylase, with cofactor vitamin B6	Levodopa-induced dyskinesias	[69, 114, 115]
Agonists of the D_1_- and D_2_-like dopamine receptors	APO-go^®^ (apomorphine)	Britannia Pharmaceuticals	Aporphine alkaloid from morphine acidification able to bind D1- and D2-like receptors, and serotonergic, adrenergic receptors	Nausea, somnolence and hypotonia	[116]

Numerous clinical trials for new treatments against AD are currently active on clinicaltrial.gov. Different research groups are testing drugs, devices, and behaviors (physical activity or different diets) to decelerate the disease or treat individual symptoms (e.g., depression, agitation, psychosis, or epilepsy). Their focus is on drugs to improve Tau protein PET imaging, brain stimulation devices, diet supplements, and physical activity to improve patients’ cognition. Most drugs aim to prevent neurodegeneration and cognitive decline [96–103]. Many groups are investigating new drugs targeting Aβ and Tau. Details on their mechanism of action and phase are listed in Table 3.

**Table 3:** Drugs against neuroinflammation currently tested in clinical trial found on ClinicalTrials.gov [103] and the ISRCTN registry

Name/phase	Features	Identifier	Duration
General immune response			
Trehalose (SLS-005)/phase II	Disaccharide able to promote autophagy and the immune response [117]	NCT05332678	2023 2025
Senicapoc/phase II	Calcium-activated potassium channel KCa3.1 inhibitor to reduce neuroinflammation [118]	NCT04804241	2022 2025
Baricitinib/phase I and II	Janus kinase inhibitor able to reduce neuroinflammation [119]	NCT05189106	2022 2024
CpG1018/phase I	Synthetic oligodeoxynucleotides that mimic bacterial DNA and trigger the immune system with unmethylated CpG dinucleotides located in CpG motifs [120]	NCT05606341	2023 2024
Microglial target			
Oligomannate (GV-971)/phase IV	Marine-derived oligosaccharide. In the gut it reconstitutes microbiota, reduce peripheral immune cells infiltration into the brain, inhibiting neuroinflammation. In the brain it prevents Aβ formation and deposition [121]	NCT05058040 NCT05181475	2021 2025
Sargramostim/phase II	Pro-inflammatory cytokine granulocyte–macrophage colony-stimulating factor, transforming microglia, reducing amyloid load, increasing synaptic area, and improving memory [122]	NCT04902703	2022 2024
AL002/phase II	Humanized IgG1 that binds to and inactivate the TREM2 [123]	NCT05744401	2023 2025
Daratumumab/phase II	Human monoclonal antibody that binds to CD38, marker of senescent microglia [119]	NCT04070378	2019 2024
Specific immune response			
Canakinumab/phase II	Monoclonal antibody against Interleukin-1β to reduce neuroinflammation [124]	NCT04795466	2021 2026
Lenalidomide/phase II	Immunomodulator increasing anti-inflammatory cytokines levels and decreasing TNF-α, IL-6, IL-8 levels	NCT04032626	2022 2024
XPro1595/phase II	PEGylated human TNF emulator lacking TNF receptor-binding activity that binds and sequesters native TNF [125]	NCT05522387 NCT05318976	2022 2025
Masitinib/phase III	Tyrosine kinase inhibitor that can accumulate in the CNS and inhibit mast cells and microglia/macrophages	NCT05564169	2022 2025
Azathioprine/phase II	Purine antagonist limiting the proliferation of lymphocytes [126]	ISRCTN14616801	2018 2024
Sargramostim/phase I	T-lymphocyte populations regulator by boosting the T-regulatory cells and suppressing T-effector function, to reduce the chronic activation	NCT05677633	2023 2024

For PD, there is a wide variety of drugs available to treat the motor symptoms of the disease, at least in the short term. The majority of treatments against PD target the dopamine system, including dopamine agonists and dopamine breakdown inhibitors (Table 2). Common dopamine breakdown inhibitors target the enzymatic activity of monoamine oxidases, to prevent dopamine breakdown in the system. A non-pharmacological treatment for PD is stereotactic deep-brain stimulation, which uses high frequency stimulation to normalize neuronal firing rates, targeting tremor, stiffness, and bradykinesias [104]. It also has beneficial effects on PD cognitive symptoms [105]. New PD treatments are being developed, including α-syn targeting therapies, neurotropic factors, cell, and gene therapies [106], as well as therapies that target the immune activation in PD. Often, immunomodulation offers a relatively simpler treatment option, utilizing preapproved drugs with known safety and tolerability profiles. Current and prospective immunomodulatory therapies can be categorized into three main groups, broad spectrum immunosuppression, specific immune pathway targeting and microglial targeting (Table 3).

## In vitro and preclinical studies for Alzheimer’s and Parkinson’s diseases and the immune system

**Fig. 3:**
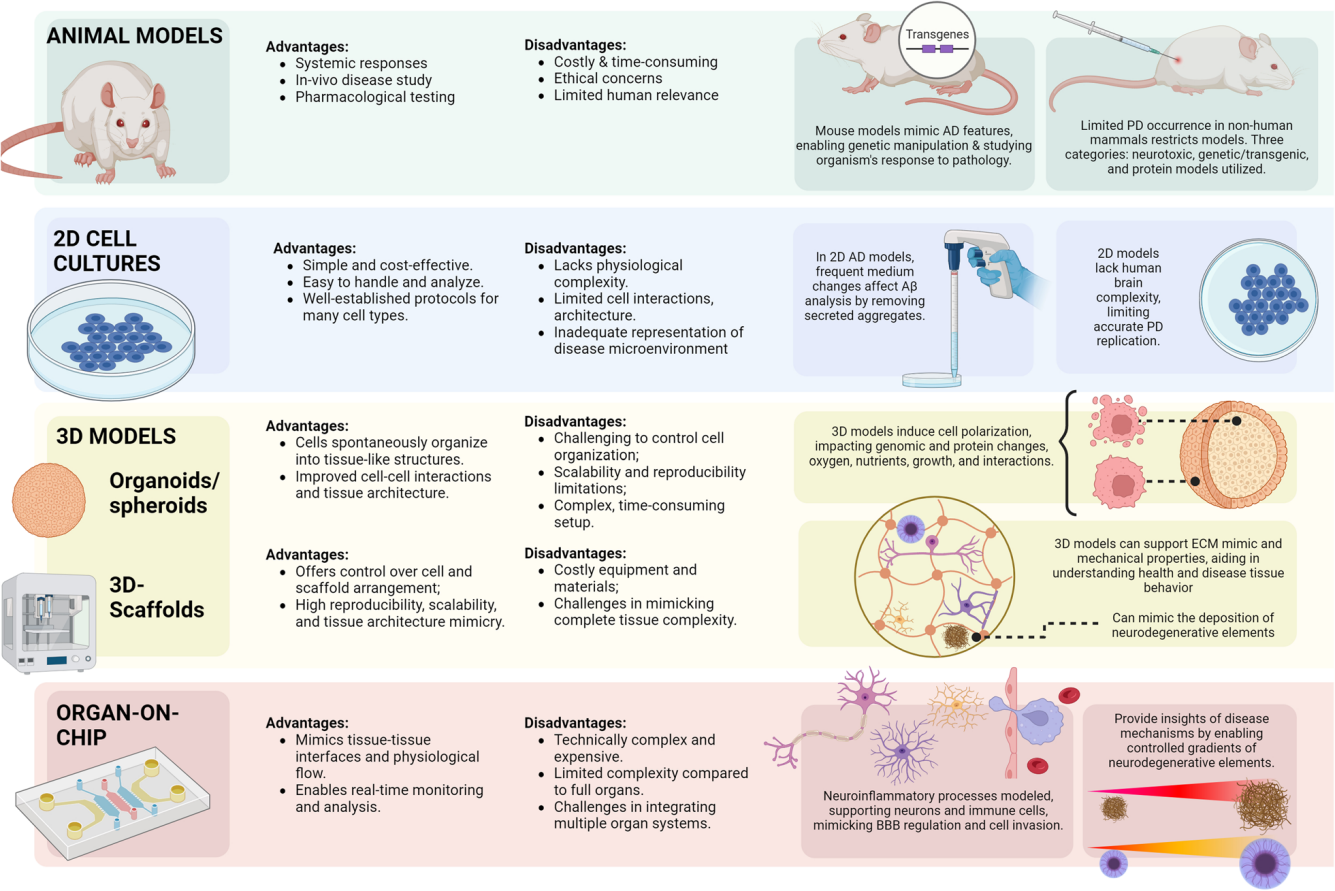
Advantages and disadvantages of in vivo and in vitro model of the neuroimmune system. The schematic shows the advantages and disadvantages of using animal models compared to cellular models, in 2D or 3D, and organ-on-a-chip. Image created with BioRender.com

In vitro and in vivo models can be used to mimic the neuroimmune systems. Even though animal models provide both physiological and behavioral processes, they do not always yield results that can be translated in preclinical drug screening for humans. Cells in 2D cultures are grown on flat surfaces that have been optimized for cell attachment and growth, whereas cells in 3D cell cultures are generally embedded inside a gel-like matrix or grown on a solid scaffold. 2D models do not mimic human brain complexity, creating a need for more physiologically relevant 3D culture formats [6–8, 12, 26, 27]. The advantages and disadvantages of both approaches are summarized in Fig. 3.

### In vitro models of the immune system

Numerous studies investigating the immune system in the context of cancer have been published research [127, 128]. The development of effective 3D models has significant implications for disease modeling and drug screening, to develop new chemotherapies or artificial tissues for transplantation [129]. T cell immunotherapy has emerged as a promising approach for treating cancer, infections, and autoimmune diseases. Jin et al. explored the latest advancements in 3D bioprinting and T cell engineering and their medical applications [130]. Among the benefits of using bioengineering approaches, other therapy applications include boosting T cell growth and differentiation into central memory-like and effector memory subsets for therapy applications. Gray et al. [131] and Snow et al. [132] discussed the potential applications of 3D bioprinting and T cell engineering in the field of neurosurgery, including creating immune chips to elucidate links between immune response and disease. Recently, 3D bioprinting has shown great potential in advancing immunotherapy, particularly in the context of natural killer (NK) cells, a type of immune cell that possess the ability to recognize and eliminate target without prior sensitization. The encapsulation of NK cells provides protection and support, allowing for their controlled release and sustained activity at the tumor site. This targeted delivery system enhances the specificity and efficacy of NK cell-based immunotherapy, minimizing off-target effects and maximizing tumor cell killing [133]. CAR-T cell therapy involves genetically modifying a patient’s T cells to express chimeric antigen receptors (CARs) that can recognize and attack cancer cells [134]. CAR-T cells cultured on a 3D scaffold have higher proliferation rate and cytokine production compared to 2D cultures. Moreover, CAR-T cells cultured on a 3D scaffold can improve tumor infiltration and antitumor activity in vivo [130]. Compared to CAR-T cells, CAR-NK cells have several advantages, including their innate ability to recognize and eliminate tumor cells without prior sensitization, lower risk of graft-versus-host disease, and potential for off-the-shelf use due to their allogeneic nature. As for CAR-T cells, 3D scaffolds can improve the survival, proliferation, and infiltration of CAR-NK cells into tumor tissues, leading to enhanced therapeutic outcomes [135]. Researchers also focused on the behavior of macrophages when cultured in 3D [136–138]. By precisely manipulating scaffold properties, such as pore size, surface topography, and mechanical cues, researchers can investigate the impact of these factors on macrophage behavior and function. Compared to 2D culture, macrophages in 3D express more cell-specific markers, even if the cells decreased their number, highlighting the influence of the scaffold microenvironment on macrophage behavior [136–138]. Bioprinted 3D scaffolds containing macrophages and antibiotics have also been used to reduce Staphylococcus aureus bacterial burdens during infections [139, 140]. This knowledge can be employed to develop innovative strategies for tissue regeneration, immune modulation, and the design of biomaterials that promote favorable immune interactions [141].

### Two-dimensional vs three-dimensional models of Alzheimer’s and Parkinson’s disease

Modeling the CNS and neurodegenerative disease in vitro can be challenging due to the complexity of the CNS, the difficulty in accessing the human brain and living primary neurons. Even if post-mortem tissues are often used, these samples represent only the final stage of the pathology and fail modeling the disease progression [6, 142, 143]. An alternative to primary cells are immortalized cell lines. The most commonly used cell line for CNS modeling is the human neuroblastoma cell line SH-SY5Y, which mimics immature catecholaminergic neurons as they express immature neuronal markers and have neurite morphology [144]. Cells can then be exposed to neurotoxins to promote the pathogenesis of AD or PD, or they can be genetically modified to overexpress APP or Tau to mimic AD [145], or α-syn to mimic PD ([146]. SH-SY5Y can also be differentiated into specific neuronal phenotypes. SH-SY5Y have some disadvantages, such as the absence of an established procedure for maintaining them in culture, which results in inconsistent experimental results and variable cell growth [6, 146, 147]. Moreover, SH-SY5Y do not exhibit electrophysiological and electrochemical features normally present in mature neurons [148]. The use of induced pluripotent stem cells (iPSC) to create reproducible models mimicking the human pathophysiology has become a rapidly developing area of research [7, 149, 150]. Somatic cells from AD and PD patients are reprogrammed followed by neuroinduction. In this case, a genetically accurate model of AD and PD is developed [6]. If the somatic cells are isolated from healthy patients, AD and PD models can be generated treating the iPSC-derived neurons in the same way as the immortalized cells [151].

#### Alzheimer’s disease

There have been several studies using 2D models created from iPSCs derived from AD patients [6, 12, 152]. The formation of abnormally phosphorylated Tau protein, increased expression of glycogen synthase kinase-3, the protein kinase that phosphorylates Tau; and upregulation of genes associated with the oxidative stress response were all achieved in a study using 2D cultures of iPSCs derived from an 82-year-old AD patient [153]. Most significantly, these 2D neuronal cultures lacked glial cell support functions, which are crucial to driving AD pathogenesis [6, 8, 12, 152]. In contrary, self-assembling peptide hydrogels seeded with neuroepithelial stem cells from hiPSCs were used in 3D studies to demonstrate the capability to simulate AD’s in vivo-like responses, such as aberrant translocation of activated P21-activated kinase and redistribution of the actin stabilizing protein drebrin, which were not observed in 2D models. The cytoskeleton dynamics depend on P21-activated kinase and drebrin, and the former is necessary for mechanotransduction pathways and AD pathology [154]. Moreover, in AD 2D models, changing the culture medium regularly can remove the Aβ secreted into the cell culture media, thus interfering with, and affecting the analysis of Aβ aggregation. 3D systems might better mimic the environment of human brain, allowing Aβ deposition and aggregation by limiting the diffusion of secreted Aβ into the cell culture medium and enabling the formation of niches that accumulate high concentrations of Aβ [6, 8, 12, 155, 156]. Additionally, human iPSCs-derived organoids can be engineered to mimic the organizational features of the human brain, e.g., the cerebral cortex to model AD. These brain organoids facilitate the pathological transfer of toxic misfolded proteins, all of which contribute to a higher translational value of brain organoids compared to that of 2D cell models. It was shown that the toxicity of Aβ oligomers was greater in 3D compared to 2D cellular models of AD [6, 8, 12]. This highlights the importance of developing complex 3D in vitro models for specific brain regions, their interactions, and local microenvironments in AD [12, 154, 155, 157]. Organoids can also be used for drug screening [158]. Lee et al., for example, isolated iPSCs from patient blood and differentiated them into neurons and astrocytes to create a 3D human AD neuro-spheroid model. This 3D model physiologically mimicked in vivo response to the drugs tested [157]. Neuronal precursors (NPCs) can also be used to resemble AD. Lomoio et al. recently developed a silk-fibroin/collagen hydrogel for the culture of patient-derived NPCs organoids with familial AD APP London mutation. The cells were cultured for 4.5 months, showing high expression of extracellular Aβ42/40 ratio deposition, and increased neuronal excitability. Moreover, cells showed a decrease in the expression of genes related to transmitter release, neural connectivity and vesicle trafficking, all established neurodegeneration pathways [159]. Kwak et al. embedded NPCs in Matrigel™ and cultured them for 6 weeks. This 3D model confirmed that Aβ42/40 can affect Tau accumulation in neuronal cells [160].

#### Parkinson’s disease

Many researchers have developed iPSC models specific to PD by reprogramming somatic cells into dopamine neurons, astrocytes, and microglia from PD patients. Complex networks of dopaminergic neurons have been developed through the use of iPSCs and organoid culture. In general, iPSC-derived neurons and organoids from familial and idiopathic PD patients replicate the main pathological disease phenotypes and are useful research tools for understanding the disease’s molecular mechanisms. Additionally, they bridge the gap between human and animal models for target validation. These 3D systems are essential because they generate intricate structures that are impossible to replicate in 2D models [7, 8, 26, 161]. In 2D models, Cooper et al. successfully produced dopaminergic neurons from human iPSCs and, after transplanting the cells into rodent brains, the treated rats showed good survival rates and improved behavior [149, 150]. The 3D model proposed by Moreno et al. used Matrigel™ and phase-guided microfluidics bioreactors differentiated human iPSCs into dopaminergic neurons. Action potential propagation along neurites and other spontaneous electrophysiological activity were reported. Accordingly, their model is reliable, economical, and exhibits biological fidelity for future use in PD modeling and drug discovery [162]. Dopaminergic neurons (DAN) derived from iPSCs were cultured in silk-based 3D scaffolds. Compared with cells in 2D, cells cultured in the scaffolds showed a dense neuronal network, exhibited high levels of α-syn and an altered purine metabolite profile [163]. Abdelrahaman et al. developed a scaffold made of self-assembled tetrapeptide. In this scaffold, DAN derived from human embryonic stem cells (hESC) responded to neurotoxins responsible for DAN loss and showed spontaneous activity over a month in culture [164]. A 3D printed scaffold made with polylactic acid (PLA), chitosan and Levodopa (a drug used to treat PD—Sect. 3) was fabricated as a potential drug delivery system. The scaffold biocompatibility was tested with human adipose-derived mesenchymal stem cells, that were alive and metabolically active over 7 days in culture. Moreover, the scaffold released the drug in PBS for 14 days, making it a suitable candidate for drug delivery [165].

### 3D models to study the crosstalk between the immune system and nervous system

Cellular communication among neighboring cells stands as an indispensable prerequisite for the preservation of tissue functions and the attainment of homeostatic equilibrium. Despite the advancements in the development of in vitro models (Sect. 4.2), modeling the interfaces between central and peripheral cells remains a challenge [6]. Interactions between neighboring cells serves as a foundational mechanism governing a multitude of critical biological processes, encompassing essential phenomena such as cell signaling, proliferation, and differentiation. Hence, crosstalk between neurons and macrophages plays a pivotal role in modulating macrophage function across diverse physiological perspectives. However, understanding of the exact mechanisms governing the equilibrium between pro-inflammatory and pro-repair responses remains inadequately elucidated. Moreover, microglia, serving as the innate immune cells of the brain, emerge as an exceedingly attractive focus for the investigation of potentially enhanced therapeutic approaches.

Traditionally, in vitro models of AD lack the ability to accurately emulate the intricate patient-specific characteristics exhibited by microglia in a 2D framework. Current in vitro models rely on the direct co-culture of neurons or glia with immune cells, lacking the structural and spatial elements present in vivo. It is therefore challenging to translate drug testing results into clinical applications due to a lack of clinical relevance or physiological intricacy in 2D neuroimmune models.

Recently developed novel 3D in vitro models of monocyte-derived microglia-like cells (MDMi) from AD patients aim to overcome these shortcomings. To simulate the neuro-glial signals present in the brain microenvironment, a co-culture platform was established with MDMi and NPC. Compared to 2D, MDMi in 3D exhibited higher survival and increased microglial marker expression, as well as mature microglial characteristics, such as highly branched morphology. Moreover, changes in cell-to-cell communication, growth factor and cytokine secretions, and amyloid-responses were seen in 3D co-cultures with neuro-glial cells [166]. Studies on the interaction between microglia and neural cells involve organoids derived from human iPSCs. In 2022, Sabate-Soler et al. fabricated midbrain organoids with dopaminergic neuroprogenitors deriving from hiPSCs. The differentiation from iPSC to neuronal progenitors was performed in 15 days, then hiPSCs-derived microglia cells were integrated into the organoids (assembloids) and cultured for 20 or 70 days. Therefore, a specific cell culture medium was optimized to allow the growth of both cell types, containing N2 supplement, 2-mercaptoethanol, IL-34, GM-CSF, BDNF, GDNF and DAPT; while TGFβ3, cAMP, and Activin were excluded as they proved toxic for microglia. After 70 days in culture, each cell type maintained its phenotype and expressed cell-specific markers. The phagocytic activity of microglia was enhanced in the assembloids, and with the cells’ removal, the assembloids size was smaller compared to organoid with neural cells only. Microglia in the assembloids also reduced the oxidative stress and the gene expression of synapsis marker, making them a good model of neuroinflammation in PD [167]. Brüll et al. implemented hiPSCs-derived astrocytes in neural organoids made of human dopaminergic neurons. The addition of astrocytes promoted the formation of a postmitotic organoid that could be used for neurotoxic and pathophysiologic studies [168]. Xu et al. differentiated hiPSCs into macrophage progenitors and NPCs to form organoids containing both cell types. Cells were then matured into macrophages and neurons. Mature organoids showed phagocytosis of apoptotic cells and reduced synapsis formation [169]. Song et al. developed organoids of neural cells from the dorsal or ventral cortex and integrated with hiPSCs-derived microglia-like cells to create a model to study the role of microglia in different neurological disorders [170]. Cai et al. developed an acoustic assembly device that can be used to form neural spheroids. Here, they formed spheroids with mice primary neurons and cultured with Aβ aggregates, showing cell death and neurotoxicity, as it happens in vivo. When microglia cells were added to the neurons and the Aβ, microglia cells migrated and surrounded the Aβ, and their activity was higher compared to organoids without Aβ, increasing the neurotoxicity [171]. Additional structural elements have been included to further enhance 3D cultures; studies have also been performed in cells cultured in 3D hydrogel scaffolds. Raimondi et al. compared semi-interpenetrating polymer networks made of type I collagen (COL) and hyaluronic acid (HA) or poly (ethylene glycol) (PEG). Cortical neurons, astrocytes and microglia from mice were co-cultured in the hydrogels for 21 days. PEG-based hydrogels led to enhanced cell metabolic activity. Furthermore, a complex network between glial cells and neurons formed. PEG-based gels also promoted synapses formation, with better results shown when cells were co-cultured in Col-PEG_2000_ cells [172]. Chemmarappally et al. developed a polyacrylonitrile/Jeffamine^®^ doped polyacrylonitrile-based fibrous scaffold and co-cultured mature neurons from neuroblastoma cell line SH-SY5Y and astrocytes from glioblastoma cell line U-87MG. After exposing the cells to PD mimicking inhibitors cells showed a higher survival rate compared to cells cultured individually on the scaffolds [173].

These studies successfully incorporated the brain-resident immune cells. It would be interesting to study the paracrine interaction between the immune system and CNS in vitro, particularly given the negative effect that cytokines can have on AD and PD progression (Sect. 2.2). Recently, to assess the role of inflammation in PD progression, Rueda-Gensini et al. co-cultured 3D bioprinted DAN in an electroconductive extracellular matrix-derived scaffold with graphene oxide, with human astrocytes and monocyte-derived macrophages, in a trans-well system. Inflammation was promoted by exposing all the cell types to an aberrant form of α-syn. DAN showed mitochondrial and synaptic dysfunctions, oxidative stress and α-syn accumulation. An increase in TNF-α, IFN-α2, Monocyte Chemoattractant Protein-1 (MCP1), IL-12p70, IL-8, IL-18 and IL-23 levels, all pro-inflammatory cytokines, was observed. The level of pro-inflammatory cytokines was higher when the immune cells were co-cultured with DA in comparison to monoculture [174].

### In vivo models for Alzheimer’s disease and Parkinson’s disease

Invertebrate and vertebrate animals have been widely used to study neurodegenerative diseases. Even if in vivo model cannot exactly replicate the complexity of the CNS, different species have been utilized to investigate single biological, molecular and pathological aspects of neurodegenerative diseases that can be translated to humans [175]. As mentioned in Sect. 4.2, studying the human brain is challenging which limits the study of neurodegenerative diseases and their progression. The use of animal models provides more flexibility and reduces these problems [176].

#### Invertebrate models

The most common invertebrate models used to study neurodegenerative diseases are the fruit fly *Drosophila melanogaster* (*Drosophila*) and the nematode worm *Caenorhabditis elegans* (*C. elegans*), because they have molecular features similar to humans. As their genome has been fully sequenced, they can easily be genetically modified. This aspect is crucial in AD modeling, as in both *Drosophila* and *C. elegance* Aβ is absent, even if APP, PSEN1, and BACE1 are expressed [175]. Thus, transgenic models of both species were developed [177, 178]. Moreover, *Drosophila* possesses forms of innate [179] and adaptive immunity [180], making it a suitable model for studying neuroimmune dynamics in AD and PD [181]. On the contrary, *C. elegance* lacks inflammatory cells, microglia, and astrocytes, meaning that only aspects unrelated to neuroinflammation can be investigated using this model [182, 183]. In *Drosophila* AD models, Aβ are surrounded by glia positive to Draper, the glia engulfment receptor, which activates the signaling pathway of Aβ phagocytose. When Draper is not expressed, locomotion problem and short life span occur [184]. By mutating the transmembrane of a homolog of the mammalian IL-1 receptor (*Drosophila* Toll gene), a decrease of the Aβ42 neuropathological activity takes place in *Drosophila*, while the gain of function of the toll receptor increases the Aβ42 neurotoxicity activity. Thus, by suppressing the Tl-NF-κβ pathway, the Aβ42’s neuropathological activity decreases [185]. Furthermore, it was shown in *Drosophila* models that the increase of ROS can lead to tauopathies progression [186–189]. Pathogenic Tau and Aβ42 also have a negative effect on behavior, the regulation of neuroinflammatory genes, and the neurodegeneration in *Drosophila*. It is interesting to note that tauopathy models showed more severe neurological impairment than amyloidopathy models [190]. *Drosophila* is also used to replicate PD. Mutants of PINK1/Parkin show impaired brain and muscle mitochondrial activity, malfunctioning flight muscle, elevated levels of oxidative stress, male sterility, low life expectancy, decreased motor function, and aberrant DAN morphology [191, 192]. Olsen et al. developed an α-synucleinopathy model to investigate the role of several genes involved in glial modifications in PD, including the ortholog of LRRK2. When these genes were suppressed, neurodegeneration, neural loss and α-syn oligomerization occurred [193].

#### Vertebrate models

A great variety of vertebrates have been used to model the AD pathology, with rodents being the most common choice for transgenic AD models and non-human primates, such as macaques, for the non-transgenic modeling of the disease [194]. Mouse models are widely used to study AD pathology, due to their ability to be genetically manipulated, mimicking the disease hallmarks and organism’s response to the pathology [195]. Transgenic models involve knock in/out of mutated genes associated with AD pathology, such APP, APOE, α-secretase and presenilins that result in an effective and early onset of Aβ accumulation or Tau hyperphosphorylation. These modifications reproduce many aspects of the pathology, such as neuronal loss [196]; synaptic dysfunction [197] and cognition impairment [198] among others. Even though transgenic mouse models are good at representing the Aβ burden, sporadic forms of the disease are considered more representative of the human condition [199]. This can be achieved using either non-transgenic mouse models—by administrating soluble Aβ oligomers or with novel methods comprising environmental challenges (e.g., chronic inflammation)—or by studying genetic variants that offer greater risk of developing AD. As stated in Sect. 2.2.1, although Aβ plaques are the central cause of the synapse loss and cognitive deficit in AD, chronic inflammation has also been implicated in the onset and progression of these AD-related pathologies [200, 201]. Novel techniques were designed to understand the role of exacerbated inflammation, by using a viral mimetic polyinosinic:polycytidylic acid (poly I:C). The poly I:C model is known to achieve an increased Aβ deposition and cognitive deficits in a non-transgenic mouse [200, 201]. Additionally, several genetic variants of TREM2 (R47H and R62H) have been associated with an increased risk of developing sporadic AD and are being studied in the AD context [202]. Despite the wide usage of the animal models, great discrepancies are observed. Most human cases occur sporadically, while most transgenic mouse models have an early onset [194]. In addition, the pro-inflammatory signaling cascades and phenotypic characteristics of immune cells in AD pathology in transgenic mice, differ from those seen in the human brain. For instance, rodent microglia tend to have a more robust and rapid pro-inflammatory response compared to human microglia. This difference can be attributed to the expression levels and functional variations of specific receptors and signaling pathways [203, 204]. These inter-species variations emphasize the importance of utilizing human models for studying and developing therapeutic interventions for AD [199].

PD does not occur naturally in any mammals except humans, so modeling the disease is limited to focusing on a single or small number of symptoms. Only 7% of PD is caused by genetic mutation [35]. Modeling the disease subtypes is more achievable, though full penetrance of the disease is also likely due to several genetic contributors, as well as environmental factors. Modeling the non-inherited forms of the disease is more complicated, and often one aspect of the disease is studied in isolation. Animal models of PD fall into three main categories, neurotoxin models, genetic/transgenic models, and protein models. Neurotoxin models involve intracerebral lesioning with neurotoxins such as 6-hydroxydopamine (6-OHDA), MPTP (1-methyl-4-phenyl-1,2,3,6-tetrahydropyridine; a prodrug of neurotoxin MPP), rotenone or paraquat. These neurotoxins can induce α-syn aggregation in the brain, as well as causing direct neurotoxicity killing DAN in the lesioned area. Interestingly, neurotoxin models are capable of inducing neuro- and peripheral infiltration [205]. A more direct in vivo model of neuroinflammation is achieved by administrating lipopolysaccharide (LPS) into the brain inducing neuroinflammation. Interestingly, LPS models also show selective DAN loss, replicating PD phenotypes and suggesting a causative role of neuroinflammation in DAN loss [206]. Genetic models of PD can replicate familial forms of the disease with known mutations in PD genes such as *PARK7, LRRK2, PRKN, PINK1* or *SCNA*. Furthermore, transgenic models can be generated to examine genes of interest thought to be involved in PD pathogenesis. Like in AD models, protein aggregation models can be generated, in PD using recombinant α-syn pre-formed fibrils based upon the prion-like transport models [207]. In vivo models allow behavioral testing to be performed. Common behavioral examinations of PD include amphetamine-induced rotation (motor function), rotarod and pole tests (coordination), gait examinations, forced swim and open field tests (anxiety, depression, and mood symptoms). Utilizing these models allows for therapeutic testing in which behavioral rescue can be used as a readout. When considering PD systemic nature, and involvement of the gut and peripheral immune system, in vivo models can offer a more complete picture of the disease than in vitro models.

### Organ-on-a-chip of neuroimmune system in Alzheimer’s disease and Parkinson’s disease

**Fig. 4:**
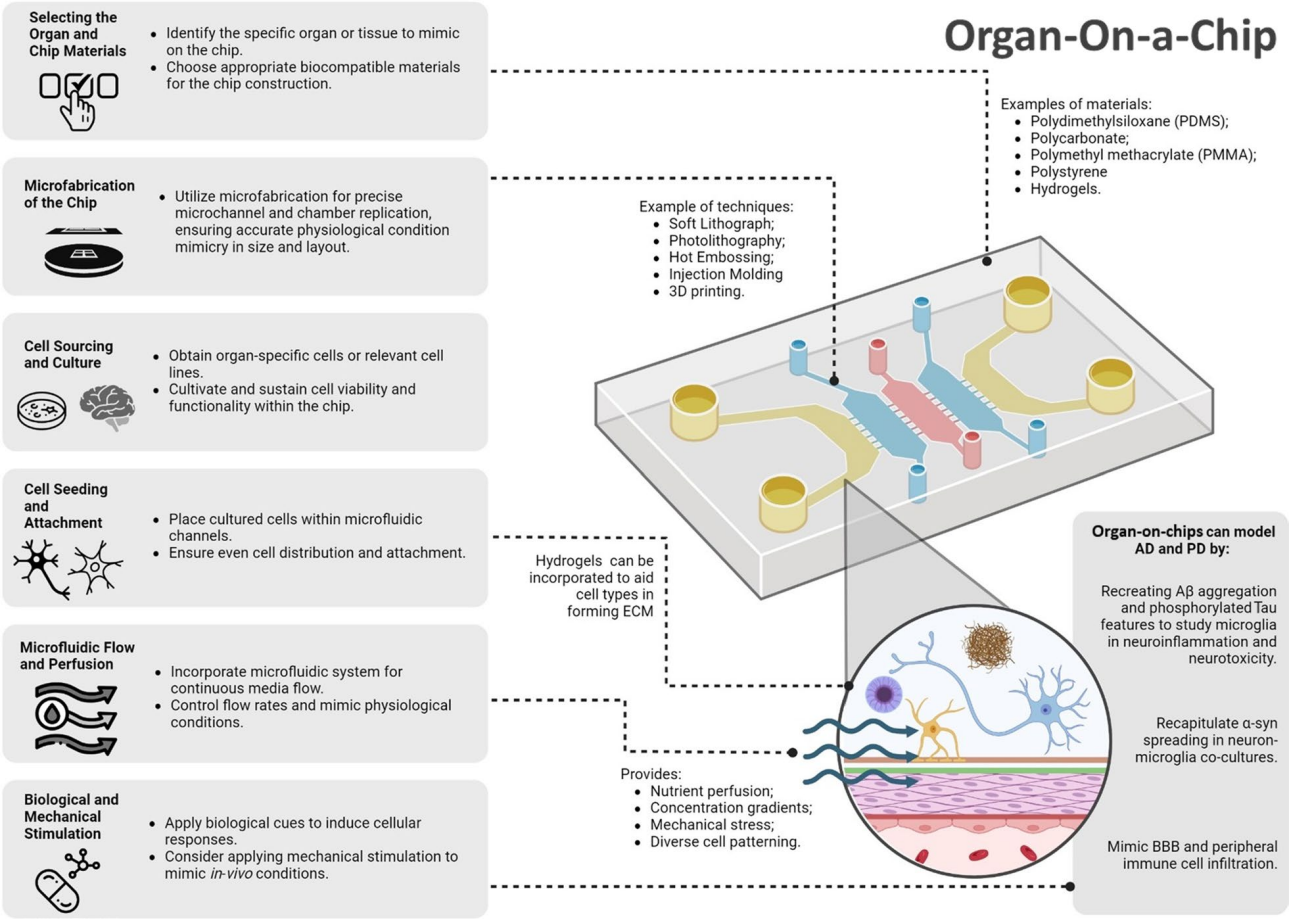
Organ-on-a-chip development: the schematic shows the steps required for developing and fabricating a microfluidic chip. Image created with BioRender.com

The emergence of organ-on-a-chip platforms has allowed for enhanced design capacities and control in building in vitro models capable of mimicking biological, biochemical, physiological, and mechanical phenomenon in living organ systems. Details on materials used for chip fabrication [208–210], hydrogels incorporated in the chips [211], and the fabrications techniques used are reviewed elsewhere [194, 212–219]. A schematic of the organ-on-a-chip development can be found in Fig. 4.

While some studies primarily focus on successful neuronal cell culture [220], there are a growing number of studies that focus on modeling more complex physiology of permeable barriers relative to the brain–immune axis. For example, a study conducted by Zhou et al. designed a blood–cerebrospinal fluid barrier microfluidic model that allowed two parallel and opposed microchannels separated by a polyester porous membrane to be continuously perfused. This allowed for the monitoring of mass transport and diffusion of molecules from each channel paving the way for further analysis into essential neuroimmune interactions within the blood–cerebrospinal barrier [221]. Similarly, a study published by Brown et al. employed a two-chamber system divided by a polycarbonate membrane made from polydimethylsiloxane (PDMS) and seeded different cell types in opposition to form their neurovascular blood brain barrier unit [222]. Salmon et al. fabricated a chip capable of co-culturing hiPSCs-derived pericytes and endothelial cells that self-assemble into vascularized networks in a temporal/spatial manner [223]. Recently, Kang et al. developed a brain-on-a-chip model, co-culturing human neurons, astrocytes, and microglia. This device was used to develop models of CNS disorders, bacterial infections, cancer, or edema, by introducing Aβ (adding cells expressing APP variants), bacterial conditioned media or bacterial toxin, cancer cells, or ammonium chloride, respectively. The device allowed for both real-time and end-point analysis [224]. Microfluidic platforms have also been used to understand the neuroinflammatory processes in AD. Park et al. developed a 3D triculture model of neurons, astrocytes, and microglia that recapitulated AD features such as Aβ aggregation and phosphorylated Tau accumulation, while also demonstrating the involvement of microglia in neurotoxic activities and neuroinflammation. The inclusion of microglial cells in the model caused significant alterations in neuronal morphology, axonal damage, and a reduction in the surface area of neurons and astrocytes, accompanied by decreased cellular density and substantial losses of both cell types. The negative impact of microglia on neurons was found to be mediated through the activation of interferon (IFN)-γ and TLR-4 signaling pathways. Inhibition of these pathways decreased the production of neurotoxic mediators and mitigated cell death [225]. Another study investigated the interaction between microglia and neutrophils, shedding light on the role of peripheral immune cells in AD neuroinflammation. The results revealed that Aβ-challenged microglia released chemoattractants that recruited neutrophils, resulting in the production of inflammatory mediators. This interaction between microglia and neutrophils contributed to neuroinflammation in AD. The findings suggest that targeting the communication between central and peripheral immune communities could be a potential strategy for alleviating immunological burdens in neuroinflammatory CNS diseases [226]. Overall, both studies provide valuable insights into cell-to-cell interactions and immune responses in AD, offering potential avenues for therapeutic interventions. A brain-on-a-chip model was used to investigate different aspects of neurovascular interactions and neurodegeneration, in two studies. The first study focused on developing a 3D in vitro model of the neurovascular unit (NVU) using six types of cells. This model successfully mimicked the brain microvasculature and demonstrated mature barrier function crucial for maintaining the integrity of the NVU. Additionally, the presence of multiple cell types in the co-culture system was found to modulate the inflammatory response in the NVU through the cytokine-mediated pathway [227]. A following study investigated the effects of particulate matter, specifically diesel exhaust particles (DEP), on neurodegeneration. The platform simulated neuro–glia–vascular interactions in a co-culture of brain endothelial cells (bECs), neurons, and glia. Exposure to DEP in the brain-on-a-chip model resulted in pathological features resembling AD, specifically, phosphorylated Tau and Aβ level changes, excessive production of hydrogen peroxide and reactive oxygen species (H_2_O_2_/ROS), and neuronal loss. Results showed that secretion of granulocyte–macrophage colony-stimulating factor by bECs, contributed to microglial activation and led to the overproduction of H_2_O_2_/ROS [228]. Jorfi et al. in 2018 cultured neuro-spheroids from engineered human neural stem cells, to overexpress APP and PSEN1, and hiPSCs in microwell array, incorporated in Matrigel™. The platform allowed neuro-spheroids to be held in place during cell differentiation. This brain-on-chip system can be used for studying AD pathogenesis and multiple drug treatments as each neuro-spheroid can be tested individually, and AD cells showed extracellular Aβ aggregates, and intracellular phosphorylated Tau [229]. The same group also assessed the infiltration of peripheral blood mononuclear cells into an AD brain 3D model made of NPC modified to overexpress Aβ, astrocytes and iPSCs-derived microglia, cultured in Matrigel™. The 3D model was cultured in the central chamber of a microfluidic chip, while different cells from the peripheral blood, namely CD8^+^/CD4^+^/CD3^+^ T cells, monocytes, and B cells, were seeded in adjacent chambers, connected by microchannels. Data suggested that the infiltration of CD8^+^ T cells into the AD 3D model enhanced microglial activation, neuroinflammation and neurodegeneration, by activating INF-γ and neuroinflammatory pathways in the glial cells [230]. A large focus of organ-on-chip platforms for the study of PD attempt to model neuroinflammation to elucidate the physiological mechanisms behind the disease. These models mainly integrate immune cells like microglia, and astrocytes that densely populate the substantia nigra while also taking into consideration infiltrating immune cells like monocytes, as well as certain subtypes of T cells as the disease progresses [76]. One of the first studies that modeled α-syn spreading in a co-cultured system of N9 microglial-like cells and H4 neuroglioma cells on-chip was conducted by Fernandes et al. In this study, a PDMS microfluidic platform was designed to house human H4 neuroglioma cells and N9 microglial cells in two chambers connected by three channels with flow being controlled with integrated pneumatic valves. This device provided the rapid spread of molecules and a regulated microenvironment, thereby imitating the process of paracrine signaling, as observed with the increase in levels of reactive oxygen in the presence of activated N9 cells [231]. Since then, organ-on-chip technologies have paved the way to study of the progression of PD with respect to the BBB. For example, Jacquet et al. examined the effect of inflammatory astrocytes on the BBB by utilizing a PD microfluidic chip model. The chip is composed of 3 lanes allowing for co-culturing endothelial-like vessels (top layer), pericytes (middle), and astrocytes (bottom layer) under perfusion to mimic the in vivo shear forces of the BBB microenvironment. This study demonstrated the same increase in blood vessel diameter as seen in human post-mortem PD brain tissue. This further highlights how inflammatory astrocytes disrupt the BBB via vascular abnormalities which may lead to PD progression [232]. Pediaditakis et al. demonstrated that astrocytes and microglia’s reactivity induced by α-syn on their substantia nigra brain-chip. Their PDMS-based model contained both a vascular channel and brain channel with seeded iPSC-derived dopaminergic neurons in addition to human primary brain astrocytes, microglia and pericytes to create a vascular–neuronal interface. Here, trehalose, a disaccharide known to decrease aggregation of proteins and neurodegeneration, had protective effects against neuroinflammation caused by α-syn, resulting in lower levels of inflammatory cytokines TNF-α and IL-6 [233]. This suggests the significance of neuroinflammation to PD pathology and progression in addition to the versatility of the organ-on-chip platform to accurately model this neuroimmune-specific behavior.

These studies collectively emphasize the importance of studying intercellular crosstalk using human cell-based models to gain insights into neurodegenerative diseases, bridging the gap between physiological and pathological aspects of the human brain. However, despite the growing research on intercellular crosstalk and human cell-based models, studies dedicated to understanding the intricate mechanisms underlying AD and PD remain relatively sparse.

## Conclusion and future directions

The intricate interactions and coordination between innate and adaptive immune cells play a pivotal role in the exacerbation of AD and PD pathology. The delicate balance between the overexpression of pro-inflammatory cytokines and the phenotypic transformation of these immune cells is a crucial factor in controlling chronic inflammation, to prevent the onset and progression of neuroimmune diseases. Nevertheless, further research is still required to unravel the complex mechanisms underlying the action and interaction of these immune cells within the context of AD and PD pathology. Modulating the function of these different immune cells holds promise for the development of better targeted and more effective treatments for AD and PD. To study AD and PD, various models have been developed, including animal models, cellular models, and more recently 3D microfluidics models. These models provide a controlled environment to investigate disease mechanisms, evaluate drug candidates, and explore the dynamic interplay between cells and tissues. However, very few in vitro studies explore the dynamic between the immune and the central nervous systems. Understanding how to modulate the immune response could reduce neuroinflammation, delay the disease progression, and eventually provide effective treatments.

Microfluidics models offer several advantages, including the ability to mimic the complex architecture of brain tissue, incorporate multiple cell types, and provide precise control over fluid flow and environmental conditions. Thus, microfluidics models in 3D enable researchers to study the intricate interactions between neuronal cells, immune cells, and the surrounding microenvironment. By using such advanced models, we can gain a deeper understanding of the disease processes and potentially identify novel therapeutic strategies. However, as stated in Sect. 4.5, there is a scarcity of studies on microfluidic chips for AD and PD. Using microfluidic devices could help to reduce the use of animal models, which are not able to provide a complete overview of human pathology. Microfluidic devices will also allow to study the paracrine interaction between immune cells and neurons, which play a key role in the progression of AD and PD.

## Data Availability

Not applicable.

## Abbreviations

AD: Alzheimer’s disease
APOE: Apolipoprotein E
APP: Amyloid precursor protein
Aβ: Amyloid beta
BBB: Brain–blood barrier
BDNF: Brain-derived neurotrophic factor
CNS: Central nervous system
DAN: Dopaminergic neurons
IL: Interleukin
Ig: Immunoglobulin
INF: Interferon
IPSCs: Induced pluripotent stem cells
MCI: Mild cognitive impairment
PD: Parkinson’s disease
PDMS: Polydimethylsiloxane
TLR: Toll-like receptor
TNF-α: Tumor necrosis factor
TREM: Triggering receptor expressed on myeloid cells
α-syn: α-Synuclein
